# Paediatric Therapeutic Development Workshop on rhabdoid tumours

**DOI:** 10.1038/s41416-026-03348-7

**Published:** 2026-02-12

**Authors:** Claudia Montiel Equihua, Jan J. Molenaar, Itziar Areso, Jaclyn A. Biegel, Patricia Blanc, Susan N. Chi, Sam Daems, Laura Danielson, Jarno Drost, Niels E. Franke, Michael C. Frühwald, Amar Gajjar, James I. Geller, Annie Huang, Pascal D. Johann, Pamela Kearns, Karsten Nysom, Suzanne O’Connor, Michael V. Ortiz, Jenny Parker, Seema Patel, Sheena Patel, Charles WM Roberts, Daniel Williamson, Joanna S. Yi, Andrew DJ Pearson, David Jenkinson, Marcel Kool, Franck Bourdeaut, Innovative Therapies for Children with Cancer (ITCC), Jan J. Molenaar, Pamela Kearns, Cancer Research UK, Laura Danielson, Sheena Patel, Cancer Grand Challenge PROTECT team, Patricia Blanc, Sam Daems

**Affiliations:** 1 LifeArc, London, UK; 2 Princess Máxima Center for Pediatric Oncology, Utrecht, The Netherlands; 3 Department of Pediatrics, Keck School of Medicine, University of Southern California, Los Angeles, CA USA; 4 Department of Pathology and Laboratory Medicine, Children’s Hospital Los Angeles, Los Angeles, CA USA; 5 Imagine for Margo, Saint-Germain-en-Laye, Paris France; 6 Dana-Farber/Boston Children’s Cancer and Blood Disorders Center, Boston, MA USA; 7 Waterland Private Equity Investments, Antwerp, Belgium; 8 I3h Institute, Université libre de Bruxelles, Brussels, Belgium; 9 Cancer Research UK, London, UK; 10 Oncode Institute, Utrecht, The Netherlands; 11 Pediatrics and Adolescent Medicine, Swabian Children’s Cancer Center, Pediatric Neurooncology, University Hospital Augsburg, Augsburg, Germany; 12 Bavarian Cancer Research Center, Augsburg, Germany; 13 Department of Pediatric Medicine, St Jude Children’s Research Hospital, Memphis, TN USA; 14 Division of Oncology, Cincinnati Children’s Hospital Medical Center, University of Cincinnati, Cincinnati, OH USA; 15 Division of Haematology/Oncology, The Hospital for Sick Children, Toronto, ON Canada; 16 The Arthur and Sonia Labatt Brain Tumour Research Centre, The Hospital for Sick Children, Toronto, ON Canada; 17 College of Medicine and Health, University of Birmingham, Birmingham, UK; 18 Innovative Therapies for Children and Adolescents with Cancer (ITCC), Paris, France; 19 Department of Pediatrics and Adolescent Medicine, Copenhagen University Hospital—Rigshospitalet, Copenhagen, Denmark; 20 Centre for Targeted Protein Degradation, School of Life Sciences, University of Dundee, Dundee, Scotland UK; 21 Department of Pediatrics, Memorial Sloan Kettering Cancer Center, New York, NY USA; 22 Cancer Research Horizons, London, UK; 23 Division of Molecular Oncology, Department of Oncology, St. Jude Children’s Research Hospital, Memphis, TN USA; 24 Translational and Clinical Research Institute, Newcastle University Centre for Cancer, Newcastle upon Tyne, UK; 25 Department of Pediatrics, Texas Children’s Cancer and Hematology Center, Baylor College of Medicine, Houston, TX USA; 26 University Medical Center Utrecht, Utrecht University, Utrecht, The Netherlands; 27 Hopp Children´s Cancer Center (KiTZ), Heidelberg, Germany; 28 German Cancer Research Center (DKFZ) and German Cancer Consortium (DKTK), Heidelberg, Germany; 29 SIREDO Oncology Center (Care, Innovation and Research for Children and AYA with Cancer), Institut Curie, Paris Cité University, Paris, France

**Keywords:** Paediatric cancer, Paediatric cancer

## Abstract

Rhabdoid tumours (RT) are malignancies of the central nervous system, kidneys, liver and soft tissues that most commonly affect very young children with survival rates below 30% in high-risk cohorts. Treatment entails surgery, intensive chemotherapy and radiotherapy, associated with substantial short- and long-term toxicities. There is an unmet need to develop targeted therapies for RT to improve patient outcomes and mitigate the toxicities of current therapy. Detailed research followed by a workshop had the objective of enabling the development of targeted therapeutics for RT. Given the inherent commonality of their biology (i.e. biallelic inactivation of *SMARCB1* or more rarely *SMARCA4*) the therapeutic approach should be similar for intra-cranial and extra-cranial tumours. DDB1–CUL4-associated factor 5 is a promising target, and the development of small molecule binders/degraders is a priority. Enhancer of zeste 2 polycomb repressive complex 2 subunit (EZH2) degraders may have greater therapeutic potential than inhibitors. Fibroblast growth factor receptor and platelet-derived growth factor receptor inhibitors may have value in subgroups. Mouse double minute 2 homologue (MDM2) is a priority target for novel therapeutic development and combination trials. Combinations of EZH2, MDM2 inhibitors and selective inhibitors of nuclear export should be evaluated robustly preclinically and drive early clinical studies.

## Introduction

Rhabdoid tumours (RT) are tumours of the central nervous system (CNS), kidneys, liver and soft tissues primarily affecting infants and young children. Around 200 patients are diagnosed across Europe and the USA each year [1].

Survival for children with RT remains very poor: the average 5-year overall survival (OS) is ~30–40% [2, 3]. Studies have revealed several molecular and clinical distinct RT subgroups and have enabled the development of risk stratification models [3]. Standard therapy typically comprises maximal safe surgery, chemotherapy and commonly radiotherapy, sometimes with myeloablative chemotherapy and stem cell rescue. Treatment is associated with substantial short- and long-term toxicities, including impairment of the developing brain in children with intracranial tumours [4]. To date most trials have divided RTs into intracranial, renal and extracranial (outside the kidney) tumours and until recently many trials have enroled relatively small number of participants. ACNS0333 was the first atypical teratoid rhabdoid tumours (ATRTs)-specific cooperative group trial and improved survival compared with historical therapies, now there is in addition the ATRT01 trial. There have been no transatlantic studies for this rare population. Thus, there is a major unmet need to develop more efficient and less toxic therapies for RT.

The first step in the introduction of a novel therapeutic into standard of care is drug development. Once developed a new therapeutic agent should undergo rapid evaluation in an early phase trial, with a dose finding or confirmation cohort and then an expansion cohort. Defining go or no-go decisions is crucial, if there is a positive signal, a confirmatory trial should be undertaken, ultimately leading to evaluation in front-line. Early dialogue with regulators, and patient advocates, is essential in the planning phase, so that early regulatory authorisation can be obtained. A coordinated global development is mandatory to avoid duplication and accelerate regulatory approval. The objective must be that trials carried out for regulatory purposes are of the maximum clinical benefit.

To facilitate the development of targeted RT therapies, reviews of the literature were undertaken, to understand the biology and epidemiology of RT, identify key drug targets and the development status of relevant drugs. Subsequently a multi-disciplinary virtual workshop with European and North American clinical and biology experts in RT, aimed to (i) review epidemiological data; (ii) share and discuss the scientific evidence for RT-associated targets; (iii) understand the tractability of targets; (iv) recommend targets for which the optimal therapeutic did not yet exist for RTs and to prioritise, based on their state of validation and tractability; (v) discuss priority drug combinations to advance into the clinical space. The objective was to develop, based on scientific evidence, an international consensus of researchers, clinicians and patient advocates of the unmet needs in RTs and prioritisation of new therapeutics. It envisioned that this consensus would be of strategic value to academic, industry, regulatory, charity and patient advocate global communities to focus resources for development.

The RT workshop was the first in the series of LifeArc Paediatric Therapeutic Development Workshops, with topics identified by an international survey of paediatric oncologists from Children’s Oncology Group (COG) and Innovative Therapies for Children with Cancer’s (ITCC) as those with the greatest unmet needs (https://www.lifearc.org/wp-content/uploads/2024/09/Unmet-needs-childhood-cancer.pdf). The workshop was organised by the Childhood Cancer Translational Challenge of LifeArc (a self-funded, not-for-profit medical research organisation and charity), ITCC (a clinical trials network focused on delivering new treatments for children and adolescents), Cancer Research UK (a medical research charity), and their translational arm Cancer Research Horizons and the Cancer Grand Challenges PROTECT team. Following the workshop, future therapeutic development of any target could progress via C-Further, the LifeArc-Cancer Research Horizons Children’s Cancer Therapeutics Consortium (https://www.c-further.org/) which provides funding and drug discovery laboratory support to de-risk early stage therapeutic projects. This article summarises the discussions and conclusions of the workshop.

## Background

Histologically, RTs are characterised by the presence of rhabdoid cells, with uncondensed chromatin, prominent nucleoli and cytoplasmic eosinophilic inclusions. RTs in the CNS are termed ATRTs and outside the CNS, most commonly in the kidney and soft tissues including the liver, neck, thorax, retroperitoneum and pelvis, extra-cranial malignant rhabdoid tumours (eMRTs) [3, 5]. ATRTs represent around 65% of all RT diagnoses, around 20% of all intracranial tumours in children below 3 years of age and are the most commonly diagnosed embryonal CNS tumour in children below 1 year [1].

Several molecular subgroups of RTs have been defined by DNA methylation and transcriptional signatures, including at least three main subgroups of ATRT: ATRT-TYR, ATRT-SHH and ATRT-MYC [6, 7]. Integration of molecular with clinical data suggest that patients with ATRT-TYR tumours have the highest 5-year OS and one study indicated that patients with ATRT-SHH and SHH and MYC tumours have a similarly inferior survival [8].This remains to be validated across different types of treatment regimens in prospective studies. Similarly, combining clinical and genetic risk factors revealed two risk-groups of eMRTs [9]. Table 1 summarises, publicly available data on epidemiology, clinical features and molecular features of the subgroups.

**Table 1:** Epidemiology, clinical features, biology and molecular features of malignant rhabdoid tumours (RT), according to molecular subgroup.

Molecular subgroup	Atypical Teratoid/Rhabdoid Tumours (ATRT)			Extracranial RT (eMRT)		
	ATRT-TYR	ART-SHH	ATRT-MYC	Rhabdoid tumour of the kidney (RTK)	Extracranial extrarenal MRT	SMARCA4-deficient extracranial RT
Location	CNS (75% infratentorial, 25% supratentorial)	CNS (65% supratentorial; 35% infratentorial) Two further two groups (SHH1 and SHH2)	CNS (50% supratentorial, 38% infratentorial, 12% spinal)	Mostly renal	Mostly extracranial, extrarenal Lesions occur in deep axial locations, extremities, cutaneous locations, visceral organs (liver most common)	Extracranial (both renal and extrarenal)
Median age at Diagnosis (months)	Most common in children <3 yrs			10.6-13 80% of patients diagnosed <2 yrs	16.8	
	12	20 Adult ATRTs reported	27 Adult ATRTs usually MYC			
Incidence/age-adjusted incidence Percentage of all ATRTs	UK: 9.5 cases/yr; US: 73 cases/yr (4 of which adult) US: 0.07 per 100,000 in 1 yr+			UK: 2.5 cases/yr US: 0.2 per million in <14 yrs	UK: 3.5 cases/yr UK/Germany: 5–5.7 per million in <1 yr; 0.1–0.2 per million at 5 yrs	5% of RT are SMARCA4-deficient
	34	44	22			
Gender difference M: F	1.3:1	1.2:1	1.08:1	1.5:1	1:1	
Presentation	Infants: lethargy, vomiting, failure to thrive. Children 3 yr + : headache, hemiplegia, head tilt, cranial nerve palsy. 25-30% present with disease seeding the spine or other brain regions.			Haematuria and/or abdominal mass. ~2/3 present with advanced disease. ~10–15% of RTK patients have synchronous ATRT at diagnosis.	Rapidly enlarging soft tissue mass; associated clinical symptoms depend on primary organ.	
**Diagnosis**	MRI of brain and spine. Lumbar CSF cytology. Renal ultrasound. Germline mutation testing. Biopsy/surgical sample to confirm diagnosis. Methylation arrays to confirm subgroup.			CT, followed by biopsy. Differential diagnosis from Wilms tumour.	CT and/or MRI, followed by biopsy.	
Standard of care	No established SOC. Depends on age and tumour location. Intensive, multimodal therapy may include: surgery, aiming for maximal safe resection (often difficult due to the invasive nature of tumour), high dose adjuvant chemotherapy: typically, induction (anthracyclines, alkylating agents, platinating agents, topoisomerase II inhibitors, anti-folates and vinca alkaloids) followed by consolidation (platinating agents, thiotepa), before maintenance alkylating agents or radiotherapy: assessed on an individual basis, avoided in children <3 yrs			No established SOC. Intensive, multimodal therapy may include: surgical resection if feasible; high dose chemotherapy (anthracyclines, alkylating agents, platinating agents, topoisomerase II inhibitors and vinca alkaloids); autologous stem-cell rescue or local radiotherapy.		
Prognosis and survival (5-yr OS), unless otherwise stated	Poor prognostic factors - younger age (<1 yr), metastasis at diagnosis,incomplete resection UK: 24% Germany: 29–32%, USA 43% 4-year OS			Poor prognostic factors - younger age, metastasis at diagnosis. UK relative survival: 22% 26-36% OS reported by SIOP, EU-RHAB	Poorer survival if RT is in liver. UK relative survival: 29% 36–50% OS reported by CHLA, EU-RHAB	
	48% (32% if <1 yr; 71% if 3 yr+)	19% Mean OS 16 mo	Undetermined; poorer than ATRT-TYR Mean OS 13 mo			
				58.3% localised disease, without loco-regional lymph node involvement. Stage 3 and 4 disease 13%-16%		
Genetics	SMARCB1 inactivation by point mutation in one allele and partial or whole loss of second allele	Compound heterozygous SMARCB1 point mutation	Homozygous, broad loss of SMARCB1. Rarely point mutation	Homozygous focal deletions, non-sense point mutations of SMARCB1	Homozygous focal deletions of SMARCB1	SMARCA4: heterozygous non-sense mutation. partial deletions leading to loss of heterozygosity. Associated with germline alterations
Methylation	Hypermethylated, particularly in promoter regions	Hypermethylated, particularly in promoter regions (to a lesser extent than TYR)	Hypomethylated Methylation analysis suggests closely related to extracranial RTs	Hypomethylated	Hypomethylated, particularly in promoter regions	
Gene expression	High expression of PRC2 complex genes such as EZH2, SUZ12, and EED. Overexpression of AURKA and HDAC1/2.			Overexpression of HOXC cluster genes, HOTAIR, BMP signalling, genes involved in kidney development	Overexpression of HOXC cluster genes, HOTAIR	
	Overexpression of melanosomal markers (e.g. TYR, MITF, DCT), ciliogenesis genes (DNAH11, SPEF1) and BMP4, EZH2, CCND1, VEGFA, ERBB2, DNMTs, HMGA2.	Overexpression of SHH (GLI2, MYCN) and Notch pathway genes (ASCL1, HES5/6, DLL1/3), CDK6, DNMTs, EZH2 and genes involved in axonal guidance and neuronal development	Overexpression of MYC, HOXC cluster genes, ERBB2.			
Master regulator TF	OTX2, MITF, LMX1A	GLI2, FOXK1, MN1	MYC, REST, RARG			
Pathway alterations	Melanogenesis pathway, tyrosine pathway, epithelial proliferation	SHH pathway, NOTCH	MYC, HOX, EZH2, DNMTs, ERBB2, mesenchymal differentiation, MAPK signalling	Cell migration, adhesion, extracellular matrix	Immune activation, inflammatory response	Cell motility, cellular movement
Cell type origin		Neural progenitors	Non-neuroectoderm			
Cytotoxic T cell infiltration	Medium	Low	High, but expressing PD-1, TIM3 and LAG3	High	High	
Immune checkpoint expression	Medium	Low	High	High	High	

WHO Blue Book; National Cancer Registration and Analysis Service (NCRAS); National Cancer Intelligence Network (NCIN).

Most information captured from literature review and discussion with attendees at Paediatric Therapeutic Development Workshop. Box left blank if no information available. [2, 3, 5, 7].

*CNS* central nervous system, *mo* months, *yrs* years.

### Current therapeutic approach

In the COG’s ACNS0333 for patients with ATRT maximal safe resection, chemotherapy, high-dose chemotherapy with autologous stem cell rescue and involved field radiotherapy resulted in 37% 4-year event-free survival (EFS) and 43% 4-year OS [2]. The majority of European countries enrol patients with ATRT into the randomised European Society for Paediatric Oncology (SIOPE) ATRT01 trial, which compares high-dose chemotherapy followed by autologous stem cell rescue to radiotherapy as a consolidation therapy (https://www.clinicaltrialsregister.eu/ctr-search/search?query=2018-003335-29). For eMRT, a standard multimodal protocol of surgery, radiotherapy and chemotherapy over 30 weeks has resulted in 72% 5-year EFS and 58.3% 5-year OS for patients with localised disease, without loco-regional lymph node involvement [5, 7]. Survival remains significantly lower for patients with stage 3 disease or with metastatic disease, with a 7% 2-year EFS and 13–16% 5-year OS [5, 7].

### Biology of malignant rhabdoid tumours

All RTs are associated with biallelic inactivating somatic mutations in the SWI/SNF subunits *SMARCB1* (>95% of cases [10, 11] or *SMARCA4* (<5% [12])). In about one third of patients with RTs there is a germline heterozygous mutation [13]. The type of *SMARCB1* mutations differs between RT subgroups. ATRT-TYR tumours mostly have point mutations in one allele accompanied by loss of the other allele via LOH of 22q. ATRT-SHH tumours usually have no LOH of 22q and display inactivating mutations in both alleles of *SMARCB1*. In contrast, in ATRT-MYC and eMRT subgroups, *SMARCB1* is most commonly inactivated by homozygous focal deletions. *SMARCA4*-deficient RTs are extremely rare entities, distinct from the others [9]. ATRT-SMARCA4 have been associated with a higher frequency of germline mutations, younger age and an inferior prognosis in comparison to *SMARCB1* mutated cases [14]. Individuals with germline-inactivating variants of *SMARCB1* or *SMARCA4* are considered to have RT predisposition syndrome (RTPS 1 and 2, respectively).

**Fig. 1:**
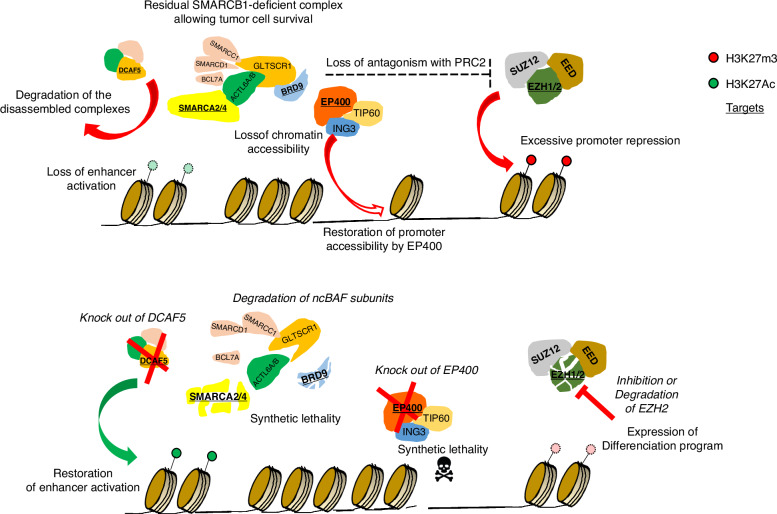
SWI/SNF complex. The SWI/SNF complex and its interactions with chromatin and other direct genetic modifiers discussed in the workshop.

SMARCB1 and SMARCA4 are subunits of SWItch/Sucrose Non-Fermentable (SWI/SNF) chromatin-remodelling complexes, which play an essential role in genome maintenance and gene regulation, controlling chromatin compaction and accessibility for transcription. The Polycomb Repressive Complex 2 (PRC2) methylates histone 3 lysine 27 trimethylation (H3K27me3), which leads to the repression of gene transcription and alters the structure of chromatin. However, SWI/SNF works to mobilise nucleosomes, in opposition to PRC2 and alter the structure of chromatin and typically leads to an open chromatin state associated with active transcription [15] Fig. 1.

SWI/SNF complexes contain core subunits: SMARCB1 (also known as SNF5, INI1, BAF47), SMARCC1 (BAF155), SMARCC2 (BAF170) and one ATPase subunit, either SMARCA4 (BRG1) or SMARCA2 (BRM), plus 6 to 11 lineage-restricted subunits [16]. Subunits interact to form three distinct configurations: canonical BAF (cBAF), polybromo-associated BAF (PBAF) and non-canonical BAF (ncBAF, or gBAF, the sole configuration remaining wholly intact within RT) [17].

SWI/SNF complexes drive the activation of developmental enhancers and bivalent promoters involved in cell fate determination and tumour suppression. Upon loss of SMARCB1, the function of SWI/SNF complexes at enhancers is substantially impaired although ncBAF complexes, of which SMARCB1 is not a component, still function. This leads to genome-wide transcriptional dysregulation that impairs cell differentiation and enables continued proliferation of progenitor cells facilitating development of RTs. The complex is still able to bind super-enhancers to enhance transcription of oncogenes [18] Fig. 1.

RTs demonstrate remarkably low mutational burden [19]; rhabdoid tumourigenesis is likely caused by the activation of oncogenic transcriptional programs in response to widespread changes in chromatin remodelling [10, 16]. Full functionality of the SWI/SNF complex can be rescued by *SMARCB1* re-expression, restoring chromatin affinity, reactivating genome-wide enhancer activation and opposing PRC2 repression at bivalent promoters [15, 18].

There is incomplete understanding of SMARCA4-deficient tumours compared to their SMARCB1-deficient counterparts, due to the rarity of these tumours and a dearth of available preclinical models and clinical data.

### Targeting the vulnerabilities of RT

As dysfunction of SWI/SNF complexes is a common biological driver of all RTs, targeted therapeutics may be applicable across subgroups, although blood-brain barrier penetrance is an additional requirement for ATRT. Targeting vulnerabilities associated with RT could benefit a wide range of additional indications as 20% of cancer types harbour mutations leading to dysregulated SWI/SNF subunits [20], and *SMARCB1* mutations are estimated to occur in 5% of all cancers [21]. However, reconstituting lost subunits would be challenging as a therapeutic intervention. SMARCA4 is a very good target in SMARCB1 deficient tumours, as the two proteins are both critical subunits of the SWI/SNF chromatin remodelling complex, and SMARCB1 loss creates a dependency on SMARCA4’s function. When SMARCB1 is deficient, SMARCA4 becomes essential for maintaining the cancer cell’s survival - a synthetic lethal vulnerability. Targeting other subunits of the SWI/SNF complex and associated vulnerabilities may represent other opportunities for targeted treatments as synthetic lethal relationships have been reported where loss of one subunit confers a dependency on another [22]^.^

Another important potential target is enhancer of zeste homologue 2 (EZH2), the catalytic subunit of PRC2 that mediates histone 3 (H3) methylation to inhibit transcription [23]. Normally antagonised by SWI/SNF, loss of SMARCB1 results in increased EZH2 activity [24]; inhibition of EZH2 in SMARCB1-deficient tumours causes tumours to shrink in vivo [25]. In the first phase I/II trial of tazemetostat, an inhibitor of EZH2 (NCT01897571), 5 of 13 (38%) adult patients with SMARCB1- or SMARCA4-deficient tumours, showed clinical benefit, including a complete response in one patient with RT [26]. In a phase I study of tazemetostat in children with relapsed or refractory RT, other SMARCB1-deficient tumours and synovial sarcoma, 14% of patients exhibited objective responses, with a 24% response in ATRT [27]. In the COG-MATCH APEC1621C trial in relapsed EZH2-mutant or SMARCB1- or SMARCA4-deficient paediatric tumours, 25% of children across all indications evaluated, including one of 12 RT patients, had disease stabilisation, suggesting tazemetostat alone does not result in adequate responses [28]. Resistance to EZH2 inhibition may arise from mutations in EZH2, but tumours may still be sensitive to other members of the PRC2 complex, such as embryonic ectoderm development (EED,) and from mutations in the retinoblastoma protein (RB1)/E2F axis, that in normal circumstances represses EZH2. RB1 inhibits cell cycle kinases (e.g. CDK2 and aurora kinase B) and inhibition of these kinases may overcome resistance to EZH2 inhibition. EZH2 inhibition may increase dependency on ATR kinase signalling, therefore combination with ATR inhibitors may be rational [29]. Secondary malignancies have been associated with EZH2 inhibitors - myelodysplastic syndrome or acute myeloid leukaemia occurred in 0.7% of 729 adults who received tazemetostat and one paediatric patient developed a T-cell lymphoblastic lymphoma and another acute lymphoblastic leukaemia. Limiting the duration of therapy may mitigate this risk, however it is uncertain if degraders would have a similar association.

Immunotherapies, such as immune checkpoint inhibitors, may demonstrate potential for RT patients. Despite a very low tumour mutational burden, ATRT-TYR, ATRT-MYC and eMRT subgroups are reported to have significant immune cell infiltration, similar to those in highly immunogenic adult tumours such as melanoma [6]. Immunotherapies are predicted to be ineffective in ATRT-SHH tumours due to their low infiltration. In a review of 251 children who had received immune checkpoint inhibitors only one of 12 (8%) with RT responded compared to 6.8% overall [30] RT robustly express B7-H3, and chimeric antigen receptor (CAR) T-cells administered intra-cerebroventricularly or intratumourally have demonstrated potent anti-tumour effects against cerebral ATRT xenografts in mice [31]. Therefore, CAR T-cells, bispecific antibodies and antibody-drug conjugate targets may be potential therapeutic approaches.

## The RT Paediatric Therapeutic Development Workshop

The workshop sought to prioritise (1) targets for which the optimal therapeutic did not yet exist for RTs (this will have medium-long term benefit to patients) and (2) combinations of drugs that were in development or already approved for other conditions and could provide a new, more immediate therapeutic option for children with RT.

### Therapeutic development pipeline and clinical trials landscape for RT

To provide context to the workshop, literature and diligence reviews were undertaken, to understand the biology and epidemiology of RT and each subgroup. Literature reviews were based on PubMed publications with the following search terms: (atypical teratoid rhabdoid tumour OR AT/RT OR ATRT) AND (therapeut* OR Target OR drug). This search returned 688 results and a small subset, determined by best match search order, were reviewed. Compared to other paediatric malignancies, there are relatively few preclinical models of RT. However, there are patient-derived xenograft, patient-derived organoid and genetically engineered preclinical models of ATRT-SHH, ATRT-MYC and eMRT that can be used to support drug discovery. ATRT-TYR samples are difficult to maintain ex vivo or in mouse models [32]. Searches in GlobalData and Cortellis pipeline databases, complemented with literature searches were carried out. For GlobalData, we considered both the Drugs and Clinical Trials databases, where AT/RT is indexed. For Cortellis, a free-text search was performed for ‘rhabdoid’. All therapeutics, clinical trials and targets were included. Twenty-one therapeutic assets currently under development for RT (Table 2) were identified. The clinical pipeline is dominated by drugs already marketed for other, primarily adult indications, with trials open to a range of cancer types that include RT, rather than specifically targeting RT biology.

**Table 2:** Drugs under development for rhabdoid tumours (RT), or for other indications but with rationale for use in RT.

Asset	Developer	Target	Modality	Mode of action	Molecular subgroup	Stage in ATRT/RT	Highest stage in any indication	Comments
Nivolumab	BMS	PD-1	Monoclonal Antibody	PD-1 inhibitor	All	Phase II	Marketed	Clear rationale for targeting RTs. Likely not effective in ATRT-SHH due to low CTL infiltration and immune checkpoint expression NCT03173950: includes a range of rare CNS cancers. NCT04416568: combines nivolumab with ipilimumab in children with SMARCB1 loss cancers
Ipilimumab	BMS	CTLA-4	Monoclonal Antibody	CTLA-4 inhibitor	All	Phase II	Marketed	Clear rationale for targeting RTs. Likely not effective in ATRT-SHH due to low CTL infiltration and immune checkpoint expression NCT04416568: combines nivolumab with ipilimumab in children with SMARCB1 loss cancers
Selinexor	Karyopharm Therapeutics	Exportin 1	Small Molecule	Exportin 1 inhibitor	All	Phase II	Marketed	Clear rationale for targeting RT NCT05985161: includes an arm for patients with rhabdoid tumours
Panobinostat	Secura Bio	HDAC	Small Molecule	HDAC inhibitor	All	Phase II	Marketed	NCT04897880: for paediatric patients, including RT and ATRT. Treatment was well tolerated; trial appears terminated due to drug supply issues.
Alisertib^a^	Puma Biotechnology	Aurora Kinase A	Small Molecule	Aurora Kinase A inhibitor	SMARCB1-deficient ATRT	Phase II	Phase II	NCT01154816: for solid paediatric tumours, including ATRT. Only 4 patients with ATRT recruited: no responses seen.
Ribociclib succinate	Novartis	CDK4/CDK6	Small Molecule	CDK4/CDK6 inhibitor	All	Phase I/II	Marketed	Clear rationale for targeting RTs. NCT05429502: solid tumours, including SMARCB1-deficient RT. NCT03434262: recurrent brain tumours, including ATRT arm
Palbociclib	Pfizer	CDK4/CDK6	Small Molecule	CDK4/CDK6 inhibitor	All	Phase I/II	Marketed	Clear rationale for targeting RTs. NCT03709680: paediatric solid tumours including rhabdoid
Cobimetinib fumarate^a^	Roche	MEK1/MEK2	Small Molecule	MAPK1/MAPK2 inhibitor	All	Phase I/II	Marketed	NCT02639546: a range of solid tumours with one patient with ATRT enroled; A/RT not in expansion cohort. No efficacy data reported.
Tazemetostat hydrobromide	Ipsen	EZH2	Small Molecule	EZH2 inhibitor	All	Phase I/II	Marketed	Clear rationale for targeting RTs. NCT05407441: Tazemetostat + ICB in children with SMARCB1 or SMARCA4 deficiency; completes 2027 NCT02601950: SMARCB1-deficient tumours including RT; RT cohort terminated early NCT02601937: SMARCB1-deficient tumours. Partial responses reported in ATRT
Paxalisib	Kazia Therapeutics	PI3Kα/mTOR	Small Molecule	PI3Kα/mTOR inhibitor	All	Phase I/II	Phase III	Clear rationale for targeting RTs. NCT06208657: planned for children with brain tumours including ATRT
Atezolizumab	Roche	PD-L1	Monoclonal Antibody	PD-L1 inhibitor	All	Phase I	Marketed	Rationale for targeting RTs. Likely not effective in ATRT-SHH due to low CTL infiltration and immune checkpoint expression Atezolizumab previously trialled as monotherapy for RT and other solid tumours; terminated due to lack of activity but single ATRT patient reported to have partial response. (NCT02541604.) NCT05286801: SMARCB1- and SMARCA4-deficient solid tumours, testing atezolizumab with anti-TIGIT antibody tiragolumab.
Tiragolumab^a^	Genentech	TIGIT	Monoclonal Antibody	TIGIT inhibitor	All	Phase I	Phase III	Clear rationale for targeting RTs. Likely not effective in ATRT-SHH due to low CTL infiltration and immune checkpoint expression Not tested as a monotherapy for ATRT. NCT05286801: SMARCB1- and SMARCA4-deficient solid tumours, testing tiragolumab with anti-PD-L1 antibody atezolizumab.
G-207^a^	Treovir		Oncolytic Virus	Targeted lysis of glioma cells	All	Phase I	Phase II	NCT02457845: open to ATRT and other supratentorial brain tumours but no ATRT patients recruited NCT03911388: phase I open to ATRT and other cerebellar brain tumours, but ATRT not included in planned phase II Drug seems focused on gliomas; presume no longer in development for ATRT
MV-NIS	Vyriad	cells expressing CD46 SLC5A5	Oncolytic Virus	Targeted lysis of CD46 high cells	All	Phase I	Phase II	Clear rationale for targeting RTs. NCT02962167 [87]: children and young adults with recurrent medulloblastoma or ATRT; completed 2023. No results reported.
B7-H3/CD19 CAR T-cells	Seattle Children’s Hospital/Cureworks	B7-H3/CD19	CAR-T	Cytotoxic T-cells expressing B7-H3 and CD19	eMRTs	Phase I	Phase I	Clear rationale for targeting RTs. NCT04483778: a number of non-CNS solid tumours, including eMRT
AGAR T-cells	Baylor College Medicine	GPC3	CAR-T	cytotoxic to GPC3 expressing cells	eMRTs	Phase I	Phase I	Clear rationale for targeting RTs.Target expressed in 50%+ eMRTs; preclinical data showed efficacy in vivo and in vitro NCT04377932 and NCT04928677: recruiting for children with solid tumours expressing GPC3
ONC-206	Chimerix	CLPP/DRD2/DRD3	Small Molecule	DRD2/DRD3 antagonist and ClpP activator	All	Phase I (adult only)	Phase I	NCT04541082: adult CNS tumours including adult ATRT
TGFBi expanded NK cells	Nationwide Children’s Hospital	Cell Therapy	NK cells		All	Phase I (planned)	Phase II	No clear rationale for targeting RTs.NCT05887882: malignant brain tumours in 1 yr+
Gallium maltolate	Imaging Biometrics	RRM1	Small Molecule	DNA synthesis inhibitor	Not specified	Preclinical	Phase I	No clear rationale for targeting RTs.Preclinical data in rat orthotopic ATRT xenograft model suggests doubling of survival time Granted FDA rare paediatric disease designation in 2024 for ATRT. NCT05887882
LP-184 (STAR-001)	Starlight Therapeutics	DNA	Small Molecule	DNA synthesis (alkylating agent)	All	Preclinical	Phase I	No clear rationale for targeting RTs. Paediatric CNS trials are planned after adult trials have begun. NCT05933265 Granted FDA rare paediatric disease designation in 2022 for ATRT
AIT-102^a^ (previously EC-8042)	OrphAI Therapeutics	DNA/SWI/SNF	Small Molecule	Displaces SWI/SNF from chromatin	All	Preclinical	Preclinical	Clear rationale for targeting RTs. Agent is analogue of natural product mithramycin, with lower toxicity than mithramycin, in development for SWI/SNF dysregulated tumours; appears to inhibit SWI/SNF activity.
CARE T-cells	Baylor College Medicine	GPC3	CAR-T	cytotoxic to GPC3 expressing cells	Extracranial RTs	Preclinical	Preclinical	Clear rationale for targeting RTs. Similar to AGAR T-cells but with IL21 expression as well as IL15 NCT02932956
Anti-IGF1R-Auristatin	Scorpius Holdings	IGF1R	peptide drug conjugate	Peptide binding to IGF1R to deliver cytotoxic payload MMAE	Not specified	Discovery	Discovery	ATRT expresses high levels of IGF1R. Preclinical testing planned in newly funded project
Anti-IGF1R-panobinostat	Scorpius Holdings	IGF1R	Peptide drug conjugate	Peptide binding to IGF1R to deliver HDAC inhibitor payload panobinostat	not specified	Discovery	Discovery	ATRT expresses high levels of IGF1R. Preclinical testing planned in newly funded project
131I-omburtamab^a^	Y-mAbs Therapeutics	B7-H3	Monoclonal antibody conjugated	Radionuclide drug conjugate. Anti B7-H3 antibody labelled with 131I and 124I	All	Inactive Previously reached Phase I/II	Inactive	NCT00089245: terminated for business reasons 2023. NCT05064306: trial open to embryonal tumours including ATRT
P-28^a^	CDG Therapeutics	TP53	Synthetic Peptide	Cell penetrating peptide which interacts with and stabilises p53	Not specified	Inactive Previously Phase I	Inactive	NCT01975116: paediatric brain tumour patients including ATRT; completed in 2015. Agent well-tolerated but no ATRT patients reported
Sendegobresib (CFT-8634)^a^	C4 Therapeutics	BRD9	Small molecule degrader		SMARCB1-deleted tumours	Preclinical/Inactive	Inactive (Phase I/II)	Drug development discontinued 2023 due to insufficient single agent efficacy NCT05355753

Where molecular subgroup is marked ‘All’, the clinical trials did not specify recruitment from any subgroup. Assets are listed in order of highest development stage for RT.

^a^Appear to be no longer in active development.

Pipeline database searches were complemented with literature searches to identify 42 targets associated with or validated in RT (Table 3). From this initial target list, 23 were identified where the optimal therapeutic did not yet exist for RTs. Professors Marcel Kool and Franck Bourdeaut (experts in the biology of rhabdoid tumours) identified six targets that did not have therapeutics in the clinic and had the highest potential for development in this indication. These six targets were the focus of discussion at the workshop.

**Table 3:** Targets with therapeutic potential for RTs, sorted by priority then alphabetical order.

Target	Molecular subgroup RT	Summary rationale and validation	Most advanced asset and development stage in RT	Most advanced asset in any indication
DCAF5^a^	All	A dependency in SMARCB1-deficient RT malignancies [40]	No drug development found	No drug development found
MDM2^a^	All	Highly expressed in all ATRT subgroups; MDM2 inhibitor decreases growth of orthotopic ATRT xenografts and prolonged survival in mice [51]	No drug development in RTs found	Brigimadlin: Phase III for liposarcoma Mavtemadlin: Phase III for endometrial cancer
SWI/SNF complex^a^	All	Loss of SMARCB1 alters SWI/SNF activity in a manner that drives pro-tumoural gene expression. Blocking SWI/SNF activity could reverse this effect. Inhibitors of SWI/SNF -chromatin binding caused tumour regression in an intramuscular rhabdoid tumour xenograft model	AIT-102—inhibitor of SWI/SNF binding to chromatin. Preclinical	AIT-102—inhibitor of SWI/SNF binding to chromatin. Preclinical
FGFR2^a^	ATRT-TYR	Upregulated in ATRT-TYR. Co-inhibition of FGFR2 and PDGFR is cytotoxic to rhabdoid cell lines	No drug development in RTs found	Several marketed FGFR2 inhibitors for adult gastric cancer and cholangiocarcinoma
PDGFRA^a^	All	Co-inhibition of FGFR2 and PDGFR is cytotoxic rhabdoid cell lines	No drug development in RTs found	Several marketed PDGFRA inhibitors for adult cancers
PDGFRB^a^	ATRT-TYR, ATRT-MYC	In vitro suppression of growth of ATRT-MYC/TYR cell lines treated with tyrosine kinase inhibitors and peripheral rhabdoid xenograft mouse model [23, 57]	No drug development in RTs found	Several marketed PDGFRB inhibitors for adult cancers
BRD9^a^	All	Rhabdoid tumour cells dependent on ncBAF complex activity, of which BRD9 is subunit [17] Depletion/inhibition of BRD9 suppresses proliferation and induces apoptosis BRD9 is incorporated into SWI/SNF in absence of SMARCB1 and is a specific vulnerability in malignant rhabdoid tumour cells [18]^,^	FHD-609 NCT0370968: Phase I SMARCB1 loss tumours (terminated) CFT-8634: NCT05355753 Phase I trial, SMARCB1 loss tumours (terminated)	CFT-8634 NCT05355753, FHD-609: a NCT03709680 PROTAC from Amphista and a BRD9 inhibitor from Rumi Scientific: all preclinical
EP400/TIP60^a^	All	Synthetic lethality between EP400/TIP60 complex and SWI/SNF complex [45]	No drug development in RTs found	No drug development found for EP400 KAT5 (TIP60 inhibitor): preclinical for solid tumours
B7-H3	All	Expressed in the prenatal brain and downregulated after birth. ATRT tumour samples and cell lines express high levels of this antigen [30]	CAR T-cells : Phase I NCT05835687	B7-H3 CAR T-cell: Phase II for adult oncology and neuroblastoma
Prioritised for combination				
EZH2	All—but ATRT-SHH may be more sensitive	A subunit of PRC2, which is antagonised by SWI/SNF complex EZH2 expression upregulated and is a dependency in tumours with SMARCB1-loss; in vivo models regress with EZH2 inhibition [25] Suggestion that role of EZH2 in ATRT may be non-enzymatic and might not be modified by EZH2 inhibitors	Tazemetostat: Phase I/II NCT05407441	Tazemetostat: marketed for follicular lymphoma and sarcoma
EED	All	A subunit of PRC2, which is antagonised by SWI/SNF complex. ORIC-944 (allosteric EED inhibitor) inhibits growth of RT xenografts [43]	No drug development in RTs found	ORIC-944: Phase I prostate cancer
Exportin 1	ATRT-TYR and others where cytoplasmic localisation seen	SMARCB1 mislocalisation to the cytoplasm has been implicated in some cases of ATRT. Nuclear localisation promoted by Exportin 1 inhibition [58]	Selinexor: Phase II NCT05985161	Selinexor: marketed for several adult cancers
Aurora Kinase B	All	May be a good combination with EZH2 inhibitor (suggested) [29]	No drug development in RTs found	AZD-2811: Phase II for SCLC
ATR	All	EZH2 inhibition may increase dependency on ATR kinase [29], May be a good combination with EZH2 inhibitor (suggested)29,	No drug development in RTs found	Ceralasertib: Phase III for NSCLC
PD-1	All—except ATRT-SHH due to low CTL infiltration and immune checkpoint expression	In xenograft model of RT, anti-PD1 antibody caused complete tumour regression in 67-80% of treated mice and prolonged survival	Nivolumab: Phase II NCT03173950 NCT04416568	Nivolumab: marketed for several adult cancers
PD-L1	ATRT-MYC, ATRT-TYR, eMRTs (trials not stratified by subgroup)	PD-L1 expression in around 50% of ATRT	Atezolizumab: Phase I/II, elicited overall response in eMRT Atezolizumab in combination with anti-TIGIT: Phase I NCT05286801	Atezolizumab: marketed for several adult cancers
TIGIT	All—except ATRT-SHH due to low CTL infiltration and immune checkpoint expression	Inhibits T and NK cells by binding to ligand poliovirus receptor (PVR) and Nectin2 on tumour cells and antigen-presenting cells. RNAseq data suggests SMARCB1/A4-deficient tumours demonstrate high expression of PVR and Nectin2	Tiragolumab: Phase I, in combination with anti-PD-L1 (NCT05286801). Development of Tirogolumab has ceased and caused NCT0528680 to close.	Vibostolimab, tiragolumab and domvanalimab: Phase III, several adult malignancies
In alphabetical order				
Aurora Kinase A (AURKA)	All	SMARCB1 (INI1) protein represses expression of AURKA in a cell type-specific manner. Knockdown of AURKA induces mitotic arrest and apoptosis in rhabdoid cell lines but not in healthy cells	Alisertib: Phase II NCT01154816 completed with 4 RT patients enroled. NCT02114229 studied single agent and combined with chemotherapy and radiation. Single agent demonstrated 1 PR and 8 SD of 30 enroled patientsNCT02114229. Combination results pending.	Alisertib: Phase II for several adult cancers Tinengotinib (Aurora kinase A and B inhibitor): Phase III for cholangiocarcinoma
NSD1	All	Mediates antagonism between SWI/SNF complex and polycomb complex. Potential for combination with EZH2 inhibitors [59]	No drug development in RTs found	No drug development found
BRD4	ATRT-MYC	BRD4 inhibitor JQ1 extends survival in ATRT-MYC model [51] Combination with EZH2 inhibitor extends survival in mouse models	No drug development in RTs found	Apabetalone: Phase III cardiovascular disease
CD47	Unclear	CD47 antibodies effective in in vivo ATRT model	No drug development in RTs found	Evorpacept: Phase III for gastric cancer Lemzoparlimab: Phase III for myelodysplastic syndrome
CDK4/6	All	SMARCB1 regulates p16, which binds to and inhibits CDK4/6. Loss of SMARCB1 leads to increased CDK4/6 activity and cellular proliferation CDK4/6 is a dependency in ATRT cells; CDK4/6 inhibitors extend survival in mouse ATRT model	Ribociclib succinate (Kisqali), Phase I/II NCT05429502	Kisqali: marketed for HER2-negative breast cancer Palbociclib, trilaciclib and abemaciclib: marketed for several adult cancers
CTLA-4	All—except ATRT-SHH due to low CTL infiltration and immune checkpoint expression	CTLA-4-expressing Treg cells found in infiltrates of RT samples	Ipilimumab: Phase II NCT04416568	Ipilimumab: Marketed for several adult cancers
DDR1	ATRT-SHH	Tumours may have dependency on DDR1	No drug development in RTs found	Dasatinib (RTK inhibitor): marketed for several adult cancers
DNMT	All	DNMT3A/3B overexpressed in RTs; inhibition of EZH2 and DNMTs causes synergistic effect to block rhabdoid tumour growth in vitro and in vivo	No drug development in RTs found	Azacitidine: marketed for several adult blood cancers
Ex vivo expanded NK cells	All subgroups of ATRT	Intra-cranial delivery of NK cells may increase anti-cancer immune response as it circumvents barriers that prevent NK infiltration into the tumour mass	NCT02271711: phase I NCT05887882: phase I planned	Phase I, in other malignancies
GD2	ATRT-MYC, ATRT-SHH	Surface expression of GD2 in ATRT is variable	No drug development in RTs found	Dinoxitumab and naxitamab: marketed for neuroblastoma
GLI1	ATRT-SHH	GLI1 inhibition by arsenic trioxide inhibits ATRT tumour growth in a mouse xenograft model Gemcitabine-induced loss of SIRT1 results in a nucleus-to-cytoplasm translocation of SHH signalling activator GLI2	No drug development in RTs found	Arsenic Trioxide, FLD-103: Preclinical
GLTSCR1^a^	Unclear	Rhabdoid tumour cells dependent on ncBAF complex activity, of which GLTSCR1 is subunit [17]	No drug development in RTs found	No drug development found
Glutamine	Unclear	Glutamine antagonist extends survival in an orthotopic xenograft model of ATRT	No drug development in RTs found	No drug development found
GPC3	eMRT	Extracranial rhabdoid tumours often express GPC3. CAR T-cells targeting GPC3 were cytotoxic to RT cells in vitro and in vivo	T-cells targeting GPC3: Phase I NCT04377932 Codrituzumab GPC3 Monoclonal Antibody: Phase I NCT04928677	AZD-5851 and BOXR-1030 CAR T-cells: Phase II for adult solid tumours ERY974 CD3/GPC3 Bispecific Antibody: Phase I for HCC
HDAC	All	HDAC inhibitor panobinostat slowed tumour growth in vivo ATRT orthotopic xenograft HDAC inhibition alone induces differentiation of eMRT cells and the combination of HDAC and mTOR inhibition is synergistic and more efficient than HDACi alone	Entinostat: Phase I/II in combination with nivolumab (NCT03838042) Panobinostat: reached phase I for RT/ATRT but discontinued due to drug supply issues NCT04897880	Belinostat, tucidinostat, valproate, romidepsin, panobinostat and entinostat: marketed for several adult cancers
IGF1R	Unclear	IGF1R highly expressed in ATRT	Two peptide-drug conjugates in discovery stage (Scorpius Holdings)	Teprotumumab: marketed for ophthalmology
JAK1	ATRT-TYR	JAK1 is overexpressed in ATRT-TYR	No drug development in RTs found	Several JAK1 inhibitors marketed for oncology, immunology and gastrointestinal indications
MEK	Unclear	MEK inhibitors selumetinib and binimetinib inhibit cell growth and induce apoptosis of AT/T cells in vitro; in vivo efficacy more limited and requires MEK inhibitor combinations	Obimetinib fumarate: reached Phase I/II with 1 RT patient treated, seems no longer in active development for RTs	Several MEK inhibitors marketed for adult cancers
MYC	ATRT-MYC, eMRT, RTK	SMARCB1 reduces MYC binding to DNA; loss of SMARCB1 could promote MYC target gene expression and oncogenesis [19]	No drug development in RTs found	Classically ‘undruggable’ IDP-121 (synthetic peptide), OTX-2002 (synthetic mRNA): Phase I/II
NURD complex	All	Antagonistic to SWI/SNF complex	No drug development in RTs found	Potential targeting with HDAC1/2 inhibitors, which are marketed, see HDAC section above
PARP	ATRT-MYC	PARP inhibitors trigger apoptosis in AT/T cell lines and prolong survival in xenografts; combination therapies being explored for other solid paediatric cancers	No drug development in RTs found	Rucaparib: marketed for metastatic castration-resistant prostate cancer, recurrent epithelial ovarian cancer, recurrent fallopian tube cancer, recurrent primary peritoneal cancer Niraparib: marketed for recurrent epithelial ovarian cancer, recurrent fallopian tube cancer, recurrent primary peritoneal cancer
PI3Kα/mTOR	Unclear	PI3Kα/mTOR inhibitor paxalisib is brain-penetrant and increased survival in a subset of mice with orthotopic ATRT xenografts	Paxalisib: Phase I/II NCT05009992	Paxalisib: Phase III for gliosarcoma and glioblastoma; Phase II for DIPG NCT03522298
Proteasome	Unclear	Proteasome inhibitors cause tumour regression in mouse ATRT orthotopic xenograft models and genetic models Neddylation inhibitors induce apoptosis in eMRT cells via a mechanism similar to proteasome inhibitors [31]	No active development in RTs found	Ixazomib: Marketed for multiple myeloma; currently in phase I/II for childhood ALL (NCT03817320) Other several proteasome inhibitors also marketed for oncology Bortezomib and carfilzomib used in paediatrics NCT02512926
RRM2	RTK, ATRT	Upregulated in ATRT and RTK. Knockdown significantly decreased cell proliferation, cell migration and induced apoptosis in ATRT cells	No drug development in RTs found	No drug development found
SHP2	RTK	RTK signalling effector. Dependency of rhabdoid cell lines on the SHP2 encoding gene PPTPN11 [53]	No drug development in RTs found	Batoprotafib, BBP-398, GH-21, JAB-3068, JAB-3312: All Phase II for NSCLC and other solid tumours in adults
SMO	ATRT-SHH	Pathway activated in ATRT-SHH	No drug development in RTs found	Sonidegib, Glasdegib, Taladegib and Vismodegib: Marketed for basal cell carcinoma and AML
VEGFR	Unknown	In ATRT biology, VEGFR2 is cross-activated following IGF1 stimulation.	No drug development in RTs found	Bevacizumab, sunitinib, sorafenib and pazopanib Several marketed VEGFR inhibitors for adult advanced epithelial ovarian, fallopian tube, primary peritoneal cancer

Assets listed are not necessarily in active development for RT and those listed are the most advanced currently in development; see Table 2 for further information about status of asset development.

^a^Target prioritised for discussion at workshop.

### Targets discussed at workshop

The targets prioritised for discussion were: (i) SMARCA4; (ii) DDB1 and CUL4 associated factor 5 (DCAF5), a cancer dependency in SMARCB1-deficient cells; (iii) BRD9 and GLTSCR1, subunits of ncBAF, the non-canonical SWI/SNF complex; (iv) EP400/TIP60, a chromatin remodelling complex; (v) mouse double minute 2 homologue (MDM2), a regulator of p53; and (vi) the receptor tyrosine kinases fibroblast growth factor receptor (FGFR) and platelet-derived growth factor receptor (PDGFR), which show increased expression in RT.

### SMARCA4

SMARCA4 inhibition has potential as a targeted treatment for SMARCB1-deficient RT as SMARCA4 can maintain the catalytic activity of the SWI/SNF complex, enabling residual activity at super-enhancer regions of the genome and driving oncogenic transcription programmes [33]. This suggests tumourigenesis in SMARCB1-deficient cells may depend on SMARCA4 presence and activity [34, 35]. Knockdown of *SMARCA4* using an inducible RNAi system impairs growth of rhabdoid cells, but pharmaceutical inhibition was detrimental for non-RT cell viability [36]. Additionally, given *SMARCA4’s* tumour suppressor properties, inhibition could conceivably result in the development of other malignancies, impeding its potential as a therapeutic target. A combination approach may overcome challenges, potentially with the simultaneous inhibition of SMARCA4 and upregulation of mutually exclusive catalytic subunit, SMARCA2.

### DCAF5

DCAF5 and other members of the DCAF family facilitate targeted ubiquitination and protein degradation by conferring target specificity to the CRL4 ubiquitin ligase complex [37, 38]. DCAF5 is required for the survival of *SMARCB1*-mutant cancers; in these tumours, DCAF5 recognises the SWI/SNF complex as misassembled and targets it for degradation [38, 39]. Inhibition, knockdown or degradation of DCAF5 causes SWI/SNF complexes to reaccumulate and, despite continued absence of SMARCB1, the residual SWI/SNF complexes retain sufficient function to mediate rapid, marked anti-cancer effects without obvious effect upon healthy cells [40, 41].

DCAF5 is thus a promising therapeutic target in RT and other cancers associated with mutations in *SMARCB1*. Although it is unclear whether DCAF5 is druggable, other members of the DCAF family have been demonstrated as druggable [42], and PROTACs and other inhibitors have been developed for WD repeat-containing protein 5 (WDR5) and EED, which share a similar β-propeller structure with DCAF5 [43, 44].

### BRD9 and ncBAF

One of three forms of the SWI/SNF complex, non-canonical BAF (ncBAF or gBAF) remains unperturbed in SMARCB1-deficient cells and may be responsible for activity at super-enhancer sites [18]. Two of its subunits, BRD9 and GLTSCR1, are incorporated into ncBAF at higher rates in SMARCB1-deficient cell lines, with BRD9 associated with loci that potentially contribute to tumourigenesis [17, 18]. Inhibiting BRD9 can deplete ncBAF function and impair survival in RT cell lines and organoids [17, 18]. Exposing RT cells to BRD9 degraders and performing a genome-wide screen could help unravel the mechanisms behind toxicity and resistance. There are four BRD9-targeted drugs currently in development, including three PROTACs and two that have reached the clinic for synovial sarcoma (Tables 1 and 2). However, the role of SMARCB1-loss and the ncBAF complex in synovial sarcoma is different from the role of these proteins in RTs, therefore the lack of efficacy observed in synovial sarcoma may not necessarily be reproduced in patients with RTs. There are substantial concerns regarding potential toxicity from and efficacy of BRD9 degradation.

Although GLTSCR1 was initially identified as a potential target (Table 3), there is no further validation evidence available in RT, and it was de-prioritised during the workshop.

### EP400/TIP60 complex

EP400/TIP60 is a chromatin remodelling complex shown to compensate for the prolonged inhibition of SWI/SNF activity by eventually re-establishing accessibility at most affected promoters. A synthetic lethality between EP400 and SWI/SNF in cancer cell lines and human cancer patients’ data was observed [45]. This compensatory relationship could represent a synthetic lethal mechanism to target in RT. One agent targeting TIP60 is currently in preclinical development (Table 3). However, since EP400/TIP60 is essential for oligodendrocyte development and myelination in the CNS, there are concerns regarding toxicity affecting myelination in mice, *Tip60* knockout is embryonic-lethal [46] and although *Ep400* knockout mice survive foetal development, they are born with CNS deficiencies [47]. However, defects in early brain development are also observed after SMARCA4 [48] and EZH2 [49], knock-out, therefore this concern may not be unique to EP400.

### MDM2

Highly expressed in all ATRT subgroups and upregulated in eMRT, MDM2 is an E3 ubiquitin ligase whose primary function is to regulate the stability of tumour suppressor p53. Comprehensive RNAi and CRISPR-Cas9 gene targeting screens identified that RT cells were proactively suppressing an otherwise functional p53 pathway to achieve a survival advantage. Since SMARCB1 loss leads to dysregulation of oncogenic pathways, inhibiting MDM2 could enable greater accumulation of p53 to compensate [50]. Inhibiting MDM2 decreases the growth of ATRT xenografts in mice, prolonging survival [51], Overall, resistance arising post-exposure to MDM2 inhibitors is not a major concern given the stability of RT genomes. There are 24 programmes for MDM2 inhibitors, although none currently indicated for RT (Table 3) and most of these have been discontinued due to thrombocytopenia,.

### Dual targeting of PDGFR and FGFR1

FGFR and PDGFR are subfamilies of receptor tyrosine kinases (RTKs), known to promote tumour growth, invasion and angiogenesis [52]. RT cells display increased expression of PDGFRα, PDGFRβ, FGFR1 and FGFR2 [53], particularly in ATRT-TYR and ATRT-MYC subgroups [32]. Employing tyrosine kinase inhibitors (TKIs) may be an effective strategy for certain subgroups: ATRT-MYC cells are sensitive to this approach, but ATRT-SHH cells are not; ATRT-TYR has not yet been tested, due to lack of models [32]. Dual targeting of FGFR1 and PDGFRα has a synergistic apoptotic effect in patient-derived RT cell lines, dependent on the activity of PDGFRα or PDGFRβ [53]. PDGFR status could provide a biomarker to stratify patients in combination therapy.

### Prioritisation of targets

RT cell lines in the Cancer Dependency Map, DepMap [54] (https://depmap.org/portal/) a suite of open-access, cancer dependency-related tools to analyse and visualise large-scale screens using CRISPR and genome-wide RNAi, may inform the prioritisation of targets. Data from DepMap and PRISM Repurposing screens at the time of the workshop demonstrated that, compared with other cancer cell lines, RT cells show higher specific dependency on (listed from higher to lower dependency): DCAF5 > EZH2 > BRD9 > SMARCA4 > PDGFRβ = FGFR1. When compared to other cancer cell lines RT cell lines have increased sensitivity to MDM2 inhibitors idasanutlin and milademetan, followed by TKIs lenvatinib and cabozantinib, with responses seemingly linked to PDGFRα status. Full loss of EZH2 has a greater impact on RT cell viability than EZH2 inhibition by tazemetostat, consistent with the hypothesis that EZH2 degradation may be more effective for RT treatment than enzymatic inhibition.

The participants at the workshop ranked the targets based on previously published methodologies [55, 56] and findings from DepMap. The greatest priority was DCAF5; followed by MDM2; 3-SMARCA4; 4-PDGFR and FGFR; 5-ncBAF (BRD9); 6-EP400/TIP60.

Additionally, although not novel, EZH2 is an important therapeutic target. Enzymatic inhibition of EZH2 has demonstrated only modest efficacy. However, alternative approaches such as degradation of EZH2 may be more effective, as RT are primarily dependent on a non-catalytic role of EZH2 in the stabilisation of the PRC2 complex, and are only partially dependent on EZH2 histone methyltransferase activity. This suggests that EZH2 enzymatic inhibitors may not fully suppress the oncogenic activity of EZH2 [57]. In addition, sustained inhibition of EZH2 is believed to be superior to acute inhibition due to short target occupancy times of current inhibitors, which presents a possible advantage for a degrader approach.

## Combinatorial strategies

### Ongoing combination trials

Immunotherapy: Since ATRT-TYR, ATRT-MYC and eMRT and RTK exhibit high immune cell infiltration, immune checkpoint inhibitors may be beneficial in patients with these subgroups^6^ and there has been case reports of activity. Trials are currently testing different combinations of checkpoint inhibitors (NCT04416568), also in combination with EZH2 inhibition NCT05407441, in patients with SMARCB1- and SMARCA4-deficient tumours (Table 2). In addition, combining a checkpoint inhibitor with a T-cell immunoreceptor with immunoglobulin and ITIM domains (TIGIT) inhibitor (Tirogolumab) was being evaluated, since there is increased expression of the TIGIT ligand in SMARCB1-deficient tumours (NCT0528680). However, the development of Tirogolumab has ceased and caused NCT0528680 to close.

Tyrosine kinase inhibitors (TKI): Vulnerabilities of RT cells to TKI, via non-canonical oncogenic mechanisms, have been consistently shown across preclinical studies [58], providing opportunities to leverage this observation in the clinic where there are potentially available drugs.

Chemotherapy: Ongoing clinical trials (Table 3) include paxalisib, a CNS-penetrant PI3Ki inhibitor, with gemcitabine (NCT06208657), as in vitro the combination is synergistic and alisertib (aurora kinase A inhibitor) chemotherapy and radiotherapy NCT02114229 although single agent phase II trials of alisertib demonstrated minimal responses in ATRT patients (NCT01154816, NCT02114229) (Table 3).

### Potential combinations with EZH2 inhibition

Based on mechanism of action, several combinations of EZH2 inhibitors with other, potentially synergistic drugs could improve efficacy.

- Selective inhibitors of nuclear export (SINE) inhibition: *SMARCB1* mutations that truncate the C-terminal region, mostly seen in ATRT-TYR tumours, can unmask a nuclear export signal, causing the truncated SMARCB1 protein to accumulate in the cytoplasm; inhibiting nuclear export with selinexor or leptomycin retains the truncated SMARCB1 protein in the nucleus and restores its tumour suppressor activity (NCT05985161) [59].
- Aurora kinase: combining an aurora kinase B inhibitor with an EZH2 inhibitor has been shown preclinically to bypass resistance [29]. Similarly, CDK2 inhibition, downstream of RB1 may overcome resistance to EZH2 inhibition [29].
- EED inhibition: like EZH2, EED is a component of PRC2. Dual inhibition could disable PRC2 activity and inhibit unrestrained cell proliferation in SMARCB1-deficient contexts where SWI/SNF cannot fulfil its usual antagonistic role [43].
- KDM2A inhibition reverses the immune-cold phenotype of NSD1-deficient tumour cells. NSD1 mediates the antagonism between SWI/SNF and PRC2 [60]; KDM2A-inhibitory compounds exist [61] but are not in drug development.
- MDM2 inhibition: in RT cell lines and PDX in vivo models, there appears to be synergy between EZH2 and MDM2 inhibition.
- ATR inhibition as EZH2 inhibition increases dependency on ATR kinase signalling [29].

### Other potential combinations

SINE inhibitor and MDM2 inhibitor has synergy in RT cell lines and PDX in vivo xenograft models, but less so in the MYC subtype xenograft model [50].

Other epigenetic modifiers: epigenetic modifiers, including DNA methyltransferase inhibitors and histone deacetylase (HDAC) inhibitors, may be promising in combination with immunomodulatory agents to target both tumour cells and the tumour microenvironment (Table 3).

Gemcitabine with SIRT1 inhibition: ATRTs, particularly the ATRT-SHH subgroup, are highly sensitive to gemcitabine in pre-clinical models, via disruption of SIRT1-mediated p53 repression. This combination could achieve a tumour-suppressing effect by acting synergistically on TP53.

### Prioritisation of combinations

There are frequently considerable challenges to obtain therapeutic in the optimal formulation for early phase clinical trials, especially if the asset is not part of regulatory submission, is late in its life cycle, or manufacturing has been discontinued. Proposed combinations should be evaluated robustly in preclinical models to determine the probability of success based on scientific evidence. This data should drive early clinical studies and would substantially aid advocating for drug access from pharmaceutical companies.

Combinations of therapeutics were prioritised by the participants of the Workshop based on biological rationale, mechanism of action (from the literature) and availability of the therapeutic. The following combinations were prioritised for evaluation in preclinical models:

i) EZH2 and MDM2
ii) MDM2 and SINE
iii) EZH2 and SINE
iv) EZH2 and EDD
v) EZH2 and aurora kinase B.

In addition, it was agreed that the hypothesis that PDGFR status could provide a biomarker to identify patients who would benefit from a combination of FGFR1 and PDGFRα inhibitors should be tested.

## Conclusion

The first Paediatric Therapeutic Development Workshop identified the urgent, hitherto unmet need for targeted therapies for RT that are more effective and less toxic than traditional chemotherapeutics and radiotherapeutics. Based on biology, therapeutic approaches should be similar for intra- and extra-cranial tumours, as dysfunction of SWI/SNF complexes is a common biological driver of all RTs. DCAF5 was identified as a promising target for drug discovery and the development of a degrader targeting DCAF5 is being progressed.

The EZH2 inhibitor tazemetostat is the first targeted therapy tested to show efficacy in some RT patients. EZH2 degradation theoretically may provide a more effective treatment than enzymatic inhibition, as EZH2 enzymatic inhibitors may not fully suppress the oncogenic activity of EZH2. The rationale for degraders is being explored further.

If there was adherence to the international consensus on minimum preclinical testing requirements for the development of innovative therapies for children and adolescents with cancer, all the future data would be generated in a harmonised manner [55].

Based on the conclusions of the workshop an international consortium has been formed to address the need to evaluate preclinically combinations of existing therapeutics, based on mechanism of action, in order that the most effective combination could be evaluated in early phase clinical trials. A unique international, trans-Atlantic consortium has been created, under the auspices of the ITCC aiming to evaluate robustly these potential combinations in preclinical in vitro and in vivo models to determine the probability of success based on scientific evidence.

This data will drive early clinical studies and will substantially aid advocating for drug access from pharmaceutical companies. Moreover, a major additional benefit of this initiative, is by bringing together international clinical trial groups, it will catalyse international interactions, resulting in more coordinated, harmonised, effective treatments, finally leading to international global collaborative trials for children with these poor outcome tumours.

The ultimate objective is to deliver new therapeutics and scientifically based combinations for children with RT.

## Data Availability

This is a commentary; no new scientific data was generated or analysed for this commentary and all data are within the paper.
